# A novel predictor of persistent ocular hypotony after pars plana vitrectomy for rhegmatogenous retinal detachment: The initial intraocular pressure difference between the eye with RRD and the fellow eye

**DOI:** 10.5935/0004-2749.2023-0326

**Published:** 2024-09-16

**Authors:** Ecem Önder Tokuç, V. Levent Karabaş, Sevim Ayça Seyyar, Ece Başaran Emengen, Ahmet Burak Güray, Kübra Atay Dinçer, Ceren Deniz Önder, Emrah Gökay Özgür

**Affiliations:** 1 Department of Ophthalmology, Kocaeli University School of Medicine, Kocaeli, Turkey; 2 Department of Ophthalmology, Gaziantep University School of Medicine, Gaziantep, Turkey; 3 Marmara University School of Medicine, Istanbul, Turkey; 4 Department of Biostatistics and Medical Informatics, Marmara University School of Medicine, Istanbul, Turkey

**Keywords:** Hypotony, Intraocular pressure, Pars plana vitrectomy, Retinal detachment, Silicone oils, Ocular hypotension, Visual acuity

## Abstract

**Purpose:**

To evaluate the predictive value of initial intraocular pressure difference of the detached and fellow eyes of patients with complex rhegmatogenous retinal detachment on postoperative persistent ocular hypotony.

**Methods:**

This retrospective observational study included 538 eyes of 538 unilateral complex rhegmatogenous retinal detachment patients with a proliferative vitreoretinopathy grade of C-1 or higher, treated with silicone oil endotamponade following pars plana vitrectomy. The patients were divided into Group A (patients having silicone oil removal without ocular hypotony; n=504) and Group B (patients with persistent ocular hypotony following silicone oil removal [n=8, 23.5%] and with retained silicone oil [n=26, 76.5%] due to the risk of persistent ocular hypotony; total n=34). Ocular hypotony was defined as an intraocular pressure of <6 mmHg on two or more occasions. Patients’ demographics, including age, sex, and follow-up time, and ocular characteristics, including ocular surgical and trauma history, initial and final best-corrected visual acuity, intraocular pressure and initial intraocular pressure difference of the detached and fellow eyes, and anatomical success rates and postoperative complications, were retrospectively collected from the electronic patient files.

**Results:**

The initial intraocular pressure was significantly lower in the detached eyes of Group B than in Group A (8.3 ± 3.5 vs. 12.9 ± 3.3, p<0.001). Also, the initial intraocular pressure difference was significantly higher in Group B than in Group A (8.9 ± 3.2 vs. 2.2 ± 2.7mmHg, p<0.001). The receiver operating characteristic curve analysis showed that the cutoff value of the initial intraocular pressure difference was 7.5mmHg for the risk of persistent ocular hypotony. The most influential factors on postoperative persistent ocular hypotony in the binary logistic regression analysis were the initial intraocular pressure difference and the need for a retinectomy.

**Conclusion:**

In eyes with complex rhegmatogenous retinal detachment treated with pars plana vitrectomy and silicone oil tamponade, the initial intraocular pressure difference could be of value in predicting postoperative persistent ocular hypotony and could guide surgeons on the decision of silicone oil removal.

## INTRODUCTION

For many years, intraocular silicone oil (SiO) has been used in vitreoretinal surgery as long-term tamponade in the management of complex rhegmatogenous retinal detachment (RRD) with proliferative vitreoretinopathy (PVR)^(1)^. The use of SiO endotamponade in complex RRD was first reported by Cibis et al.^(2)^ in 1962, and later studies have supported its role in improving RRD treatment success rates due to its chemically inert nature and long-term durability^(3^,^4)^.

Ocular hypotony is defined as intraocular pressure (IOP) <6 mmHg.^(5)^ Ocular hypotony becomes chronic or persistent after ocular trauma, glaucoma surgery, chronic uveitis, or RRD with PVR^(5)^, with lasting and devastating consequences, including hypotony maculopathy, vision loss from optic neuropathy, and end-stage complications (e.g., phthisis bulbi)^(6^,^7^,^8^,^9)^. Intraocular SiO injection is a well-established management strategy for persistent ocular hypotony^(10)^. However, prolonged SiO tamponade results in complications, such as emulsification, band keratopathy, increased IOP, cataracts, and silicone-related vision loss^(11^,^12)^. As an alternative, topical ibopamine eye drops, intravitreal therapies (e.g., corticosteroids, gases, and ophthalmic viscoelastic devices [OVDs]), ultrasonic biomicroscopy-assisted endoscopy, and intraocular silicone balloon implants have also been used to increase IOP in persistent ocular hypotony^(10^,^13^,^14^,^15^,^16^,^17)^.

Intravitreal SiO may be used in the long-term on a case-by-case basis to avoid the devastating complications of persistent ocular hypotony. Preoperative ocular hypotony, preoperative ciliary body detachment, the presence of PVR, a long axial length, a history of trauma, multiple previous surgeries, and retinectomy are among the risk factors for persistent ocular hypotony after SiO removal in patients with RRD^(11^,^12^,^13^,^14^,^15^,^16^,^17^,^18)^. Therefore, it is essential to predict patients who may develop persistent ocular hypotony, especially after complex RRD surgeries.

Although preoperative ocular hypotony is already known to be a risk factor for persistent ocular hypotony^(5)^, to the best of our knowledge, the difference in the lOP between the affected (RRD) eye and the unaffected fellow eye of patients with RRD has not been evaluated. We hypothesized that the initial intraocular pressure difference (IOPD) at presentation between the affected and unaffected eyes of patients with RRD might predict postoperative persistent ocular hypotony, even without actual initial ocular hypotony in the affected eye. Therefore, in this study, we aimed to evaluate the preoperative predictive factors for persistent ocular hypotony in patients with RRD with a PVR grade of C-1 or higher and treated with pars plana vitrectomy (PPV) and SiO injection, with particular focus on the initial IOPD.

## METHODS

### Study design

This single-center, observational, retrospective cohort study included all unilateral complex patients with RRD treated with SiO injection following PPV for a PVR grade of C-1 or higher^(19)^ and who underwent at least 6 months of follow-up between January 2011 and June 2021 at the Kocaeli University School of Medicine Hospital, Kocaeli, Turkey. The patients were identified by reviewing electronic health records and the demographic, ophthalmological, and surgical data retrospectively collected from the records.

### Ethical considerations

The study protocol was approved by the Institutional Review Board of the Kocaeli University School of Medicine, and the study was conducted according to the tenets of the Declaration of Helsinki. All patients or their legal representatives provided written informed consent to participate and have their medical information used in the study at their presentation, following the routine protocol of the Kocaeli University School of Medicine.

### Data collection

The patients were divided into two groups, A and B. Group A consisted of patients having uneventful SiO removal without any postoperative ocular hypotony, while Group B included patients with persistent ocular hypotony following SiO removal and patients with SiO retained due to the risk of postoperative persistent ocular hypotony. Persistent ocular hypotony was defined as IOP <6 mmHg on two or more occasions. SiO retainment was defined as eyes with a peripapillary choroidal fold under intraocular SiO, IOP <6mmHg under intraocular SiO, reinjection of SiO due to ocular hypotony after SiO removal surgery, and inoperable RRD and ocular hypotonic findings under intraocular SiO. In addition, SiO was not removed from the eyes of patients with peripapillary choroidal folds and additional hypotonic maculopathy and/or optic nerve swelling on optical coherence tomography images, even if IOP >6 mmHg. Topical steroids were administered hourly in all patients with retained SiO.

Patients with known glaucoma or presenting with lOP >21 mmHg in any eye, a history of previous vitreoretinal surgery for any diagnosis, a history of intraoperative ciliary body detachment, vitrectomy in combination with encircling or segmental scleral buckle surgery, and treatment with endotamponade other than SiO were excluded from the study.

The demographics collected were the patients’ age, sex, and follow-up time. The comprehensive preoperative and postoperative ophthalmological data of both eyes of the patients included results of the best-corrected visual acuity (BCVA) assessed with an electronic Snellen chart, slit-lamp biomicroscopic evaluation, dilated fundus examination, and IOP assessment (the mean of three repeated measurements) with Goldman applanation tonometry or the Tono-Pen AV1A tonometer (Reichert Inc., Depew, NY, USA). In addition, specific RRD-related data of the affected eye included previous ocular surgical and trauma history, the presence of high myopia (refractive error < -6.0 diopters), the retinal tear count, the need for retinectomy, the degree of retinectomy (if any), additional intraoperative procedures during initial surgery (if any), the need for revision surgery, procedures during revision surgery (if any), and postoperative complications. The IOPD between the affected eye and the unaffected fellow eye was calculated by subtracting the IOP of the affected eye from that of the fellow eye.

### Surgical procedures

All patients underwent standard 3-port, 23-gauge PPV, performed by a single surgeon (author VLK), using the OS4 device (Oertli Instrumente AG, Berneck, Switzerland) equipped with the Resight noncontact wide-angle viewing system (Carl Zeiss Meditec, Germany). The surgical procedure included posterior hyaloid detachment (if not already detached), perfluorocarbon injection, detailed vitreous base shaving, and fluid-air exchange. First, the subretinal fluid was drained through the most posterior retinal tear without drainage retinotomy, and the retinal tears were treated with endolaser photocoagulation. Next, the eyes were injected with purified 5000 cst SiO endotamponade (Teknomek, Istanbul, Turkey) through air-SiO or perfluorocarbon-SiO exchange. SiO removal was performed with the same 23-gauge PPV and Resight noncontact wide-angle viewing system, while maintaining the IOP using a balanced salt solution. After complete SiO removal, fluid-air exchange was performed at least three times to ensure thorough removal of SiO droplets from the vitreous cavity. Additional intraoperative procedures during PPV included pars plana lensectomy or phacoemulsification with intraocular lens implantation (if the patient also had cataracts), internal limiting membrane peeling, epiretinal membrane peeling, relaxing retinotomy, and retinectomy, if required.

### Statistical analysis

SPSS Statistics version 20.0 for Windows (IBM Corp., Armonk, NY, USA) was used for statistical analysis. First, the data normality assumption was assessed using the Kolmogorov-Smirnov test and histograms. Next, dependent and independent nonparametric continuous variables were compared using the Mann-Whitney *U* test and the Wilcoxon test, respectively. Categorical variables were compared with the Pearson chi-square or Fisher’s exact test, as well as the McNemar test in the case of dichotomous repeated measures. Continuous data were represented as the mean ± standard deviation, while categorical data were summarized as counts (percentages), unless otherwise noted.

The BCVA obtained in Snellen fractions was converted to the logarithm of the minimum angle of resolution (logMAR). The factors significantly associated with persistent postoperative ocular hypotony in the univariate analysis were also assessed with binary logistic regression analysis to determine the most influential ones. Furthermore, receiver operator characteristic (ROC) analysis was performed to determine whether the variables of interest could be used as a diagnostic test. A two-sided p-value of <0.05 was considered statistically significant.

## RESULTS

In total, 538 eyes of 538 patients were treated with PPV and SiO endotamponade for unilateral complex RRD with a PVR grade of C-1 or higher during the study period. The mean follow-up time of the patients was 27.6 ± 15.4 (range: 10-129) months. Of the 538 eyes, 504 (93.7%) had uneventful SiO removal (Group A), with a mean SiO endotamponade duration of 8.8 ± 4.8 (range: 3-36) months, while 34 (6.3%) eyes (Group B) either needed to have SiO retained due to the risk of postoperative persistent ocular hypotony (n = 26, 76.5%) or had postoperative persistent ocular hypotony after SiO removal (n = 8, 23.5%). Tables 1 and 2 show the baseline and perioperative characteristics of the patients, respectively. In addition, in univariate analysis, the extent of RRD, the need for retinectomy, and redetachment rates were significantly higher in Group B compared to those in Group A (Tables 1 and 2).

**Table 1:** Baseline characteristics of patients included in the study

	Overall Cohort (*N*=538)	Group A (*n*=504)	Group B (n=34)	p-value^†^
Age, years				
Mean ± standard deviation	57.8 ± 13.8 (10-81)	58.0 ± 13.3 (15-81)	55.1 ± 20.2 (10-77)	0.433^‡^
Range				
Sex, n(%)				
Female	190 (35.3)	177(35.1)	13 (38.2)	0.713^§^
Male	348 (64.7)	327 (64.9)	21 (61.8)	
High myopia*, n (%)	91 (16.9)	86 (17.1)	5 (14.7)	1.000^‖^
Previous cataract surgery, n (%)	22 (4.1)	20 (4.0)	2 (5.9)	0.642^‖^
Previous ocular trauma, n (%)	46 (8.5)	40 (7.9)	6 (17.6)	0.060^‖^
Lenticular status, n (%)				
Phakic	298 (55.4)	281 (55.8)	17(50.0)	0.772^§^
Pseudophakic	236 (43.9)	219 (43.5)	17(50.0)	
Aphakic	4 (0.7)	4 (0.8)	0 (0.0)	
Extent of RRD, n(%)				<0.001^§^
<180°	188 (34.9)	160 (36.9)	2 (5.9)	
≥80°	350 (65.1)	330 (63.1)	32 (94.1)	
Retinal tear count, n(%)				
Single tear	228 (42.4)	215 (42.7)	13 (38.2)	0.613^§^
Multiple tears	310 (57.6)	289 (57.3)	21 (61.8)	
Retinal dialysis, n (%)	8 (1.5)	6 (1.2)	2 (5.9)	0.086^‖^

RRD= rhegmatogenous retinal detachment. * Less than -6.0 diopters. ^†^ Group A vs. Group B. ‡ Mann-Whitney *U* test. ^§^ Pearson chi-square test. ^‖^ Fisher’s exact test. The bold value indicates statistical significance.

**Table 2:** Perioperative characteristics of patients in group A and group B

	Group A (n=504)	Group B (n=34)	p-value
Follow-up, months			0.637*
Mean ± standard deviation	27.6 ± 15.7	26.4 ± 9.6	
Range	(10 - 129)	(16-46)	
Retinectomy, *n* (%)	53 (10.5)	9 (26.5)	0.010^†^
Extent of retinectomy, n (%)			0.066^†^
<180°	27 (49.1)	1 (11.1)	
≥180°	25 (50.9)	8 (88.9)	
Combination with phacoemulsification, n (%)	76 (15.1)	6 (17.6)	0.627^†^
Redetachment, n (%)	45 (8.9)	8 (23.5)	0.012^†^
Complications, n (%)			<0.001^‡^
Ocular hypertension	29 (19.5)	0 (00.0)	
Macular edema	20 (13.4)	0 (00.0)	
Cataract	40 (26.8)	0 (00.0)	
Silicone oil in the anterior chamber	13 (8.7)	0 (00.0)	
Corneal haze	2 (1.3)	3 (15.0)	

* Mann-Whitney *U* test. ^†^ Fisher’s exact test. ^‡^ Pearson chi-square test. Bold values indicate statistical significance.

### IOP

The mean IOP at presentation of the affected and unaffected fellow eyes of all patients at presentation was 12.6 ± 3.5 (range: 3-21) mmHg and 15.2 ± 2.7 (range: 9-21) mmHg, respectively (p<0.001), with a mean IOPD of 2.6 ± 3.2 (range: -7 to - 13). Of the 538 eyes, 7(1.3%) showed ocular hypotony at presentation and 34 (6.3%) at the final visit (p<0.001). All eyes with initial ocular hypotony (n = 7, 1.3%) also had SiO retainment due to the risk of postoperative persistent ocular hypotony at the last examination. However, of the 34 eyes in Group B, 27 (79.4%) initially had IOP ≥6. The IOP at presentation and the final IOP in the affected eye were significantly lower, and the IOPD between the affected eye and its unaffected fellow eye at presentation was significantly higher in Group B than in Group A (Table 3).

**Table 3:** IOP and IOPD in afected and unafected fellow eyes of patients

	Group A (*n*=504)	Group B (*n*=34)	p-value^‡^
Affected Eye			
Presenting IOP,			**<0.001**
Mean ± standard deviation	12.9 ± 3.3	8.3 ± 3.5	
Median (IQR)	13.0 (4.0)	8.0 (3.3)	
Final IOP			<0.001
Mean ± standard deviation	14.9 ± 3.2	9.1 ± 3.1	
Median (IQR)	15.0 (4.0)	9.0 (3.3)	
p-value*	<0.001	0.021	
Fellow Eye			
Presenting IOP,			<0.001
Mean ± standard deviation	15.0 ± 2.7	17.2 ± 1.9	
Median (IQR)	15.0 (4.0)	17.0 (2.3)	
Final IOP			<0.001
Mean ± standard deviation	15.1 ± 2.8	18.0 ± 1.8	
Median (IQR)	15.0 (4.0)	18.0 (2.3)	
p-value*	0.964	0.001	
IOPD			
At Presentation			<0.001
Mean ± standard deviation	2.2 ± 2.7	10.2 ± 1.1	
Median (IQR)	2.0 (4.0)	10.0 (1.3)	
At Final Visit	0.2 ± 3.2		<0.001
Mean ± standard deviation	0.0 (4.0)	8.9 ± 3.2	
Median (IQR)		9.0 (2.0)	
p-value*	<0.001	0.138	

IOP= intraocular pressure; IOPD= intraocular pressure difference; IQR= interquartile range. * Wilcoxon signed-rank test. ^†^ Mann-Whitney *U* test. Bold values indicate statistical significance.

**Figure 1:**
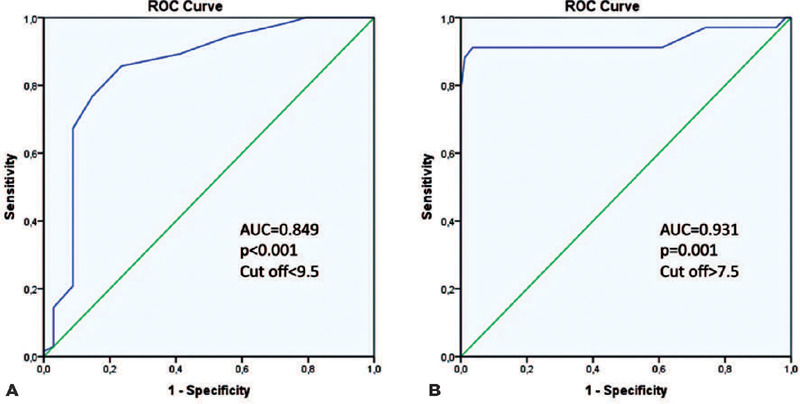
ROC analysis of ocular hypotony according to the initial IOP of the afected eye **(A)** and the IOPD **(B)**. **(A)** Patients with initial IOP <9.5 mmHg were more likely to have postoperative ocular hypotony, with 85% sensitivity and 77% specifcity. **(B)** Patients with initial IOPD >7.5 mmHg between the afected eye and its unafected fellow eye were more likely to have postoperative persistent ocular hypotony, with 91% sensitivity and 96% specificity.

ROC analysis of the initial IOP in the affected eye revealed the optimal cutoff with the highest sensitivity (85%) and specificity (77%) values at a raw score of 9.5mmHg, with an area under the curve (AUC) of 0.849 (95% confidence interval [CI] 0.767-0.931; p<0.001), as shown in Figure 1A. ROC analysis of the initial IOPD between pairs of affected and unaffected fellow eyes revealed the optimal cutoff with the highest sensitivity (91%) and specificity (96%) values at a raw score of 7.5, with an AUC of 0.931 (95% CI 0.856-1.000; p=0.001), as shown in Figure 1B.

AUC= area under the curve; IOP= intraocular pressure; IOPD= intraocular pressure diference; ROC= receiver operator characteristic.

Binary logistic regression analysis showed that the effects of the extent of RRD, retinectomy during PPV, redetachment after the first surgery, the IOP of the affected eye at presentation, and the IOPD between the pairs of eyes were statistically significant (Χ^2^_5_ =169.54, p<0.001). The analysis explained 71.9% (Nagelkerke *R^2^*) of the variance in postoperative persistent intraocular hypotony and correctly classified 98.7% of the cases, with 85.3% sensitivity and 99.6% specificity. In addition, the analysis showed that an increasing initial IOPD (p<0.001) and the presence of retinectomy (p<0.001) during PPV were the most influential factors predicting postoperative persistent intraocular hypotony (Table 4).

**Table 4:** Binary logistic regression predicting the likelihood of postoperative ocular hypotony

	*B*	SE	Wald	p-value	OR	95% CI for OR	
						Lower	Upper
Initial IOP	-0.13	0.13	0.99	0.319	0.88	0.68	1.13
Initial IOPD	-1.18	0.19	36.05	**<0.001**	0.31	0.21	0.45
Extent of RRD	0.81	0.99	0.68	0.410	2.26	0.32	15.85
Retinectomy	-2.60	0.84	9.51	**0.002**	0.07	0.01	0.39
Redetachment	0.31	0.88	0.13	0.772	1.36	0.24	7.7
Constant	11.35	2.47	21.13	**<0.001**	85011.12		

*B*= unstandardized regression weight; SE= standard error; CI= confidence interval; IOP= intraocular pressure; IOPD= intraocular pressure difference; OR= odds ratio; RRD= rhegmatogenous retinal detachment

### Best-corrected visual acuity

The BCVA of all patients significantly improved from 1.58 ± 0.98 logMAR (Snellen equivalent ~20/800; range: 0.05-3.00 [Snellen equivalent ~20/25-20/25000]) at presentation to 0.55 ± 0.53 logMAR (Snellen equivalent ~20/63; range: 0.00-3.00 [Snellen equivalent ~20/25-20/25000]) at the final visit (p<0.001). This significant improvement was also observed when Group A (from 1.54 ± 0.97 logMAR [Snellen equivalent ~20/800] to 0.50 ± 0.43 logMAR [Snellen equivalent ~20/40], p<0.001) and Group B (from 2.23 ± 0.87 logMAR [Snellen equivalent ~20/2500] to 1.41±0.96 logMAR [Snellen equivalent ~20/500], p<0.001) were evaluated separately. However, the BCVA at presentation (2.23 ± 0.87 logMAR [Snellen equivalent ~20/2500] vs. 1.54 ± 0.97 logMAR [Snellen equivalent ~20/800], p<0.001) and the final BCVA (1.41 ± 0.96 logMAR [Snellen equivalent ~20/500] vs. 0.50 ± 0.43 logMAR [Snellen equivalent ~20/40], p<0.001) were significantly worse in Group B than in Group A. The BCVA at presentation and the final BCVA of all patients were categorized into <20/200 Snellen equivalent (>1.00 logMAR; finger counting, hand motion, and light perception), >20/200 Snellen equivalent (≤1.00 logMAR), and ≥20/40 Snellen equivalent (≤0.30 logMAR) and are shown in Table 5 for the overall cohort, Group A, and Group B. Although the proportion of patients in the <20/200 BCVA category significantly decreased, the ratio of patients with BCVA ≥20/200 and BCVA ≥20/40 significantly increased in the overall cohort and Group A. The proportion of patients with BCVA ≥20/200 also increased in Group B; however, although the proportion of patients with BCVA = 20/200 decreased and that of patients with BCVA = 20/40 increased in Group B, the change was not statistically significant (Table 4).

**Table 5:** BCVA at presentation and the fnal BCVA of patients according to categories

BCVA Categories	Overall Cohort (n=538)	Group A (*n*=504)	Group B (*n*=34)	p-value^†^
<20/200 Snellen equivalent^*^, n(%)				
At presentation	378 (70.3)	348 (69.0)	30 (88.2)	0.019
At final visit	112 (20.8)	88 (17.5)	24 (70.6)	<0.001
p-value^†^	**<0.001**	**<0.001**	0.109	
≥20/200 Snellen equivalent, n(%)				
At presentation	201 (37.4)	197 (39.1)	4 (11.8)	0.001
At final visit	460 (85.5)	452 (88.3)	18 (52.9)	<0.001
p-value^†^	**<0.001**	**<0.001**	**0.002**	
≥20/40 Snellen equivalent, *n* (%)				
At presentation	48 (8.9)	48 (9.5)	0 (0.0)	0.061
At final visit	236 (43.9)	233 (49.6)	3 (8.8)	<0.001
p-value^†^	<0.001	<0.001	0.250	

BCVA= best-corrected visual acuity. * BCVA of finger counting, hand motion, and light perception^†^ McNemar test^‡^ Comparison of Group A and Group B with Fisher’s exact test. Bold values indicate statistical significance.

## DISCUSSION

Persistent ocular hypotony after vitreoretinal surgery is a well-established complication. Despite an anatomically reattached retina, persistent ocular hypotony can lead to poor vision and even phthisis bulbi, disappointing both patient and surgeon alike. Although the relationship between a low preoperative IOP and postoperative persistent ocular hypotony is clear, this study addressed a previously unreported issue, that is, the initial IOPD between affected and unaffected fellow eyes, to predict postoperative persistent ocular hypotony. In the preoperative period, IOPD ≥7.5 mmHg between the patient’s eyes was determined as the warning threshold for postoperative hypotony. The preoperative mean IOP was lower in the operated eye compared to the unaffected fellow eye (8.3 vs. 12.9 mmHg) in patients with postoperative persistent ocular hypotony. In Group B (n=34), 7 eyes were hypotonic, 27 were nonhypotonic preoperatively, and none of the fellow eyes were hypotonic. The mean IOPD between fellow eyes was ~10mmHg in patients who developed postoperative permanent ocular hypotonia. Based on the ROC analysis results, the cutoff value of the preoperative IOPD between two eyes was 7.5 mmHg, with 91% sensitivity and 96% specificity, in patients who developed postoperative persistent ocular hypotony. However, better-grouped comparative studies with fewer subgroups are needed to clarify the numerical accuracy and sensitivity/specificity of this cutoff value.

The IOPD between the two eyes is usually 2-3 mmHg. Various factors, such as decreased aqueous secretion and increased uveoscleral outflow due to ciliary body edema and RRD, the formation of the third subretinal space, and reabsorption of the retinal fluid by the retinal pigment epithelium, have been implicated in a lowered IOP before surgery^(5)^. Surprisingly, our findings showed that both preoperative and postoperative IOP values of fellow eyes were higher in the postoperative hypotonic group than in the postoperative normotensive group. IOPD between the fellow eye with a normal but relatively higher IOP and the affected eye as greater in the postoperative hypotonic group than in the postoperative normotensive group, which was associated with an increased risk of postoperative ocular hypotonia after RRD. Thus, even when the IOP is within normal limits, it does not always indicate correct functioning of the mechanism that maintains the IOP. The high IOPD between the unaffected fellow eye and the affected eye should suggest the possibility of relative ocular hypotony in the affected eye. Our findings showed the presence of relative hypotonia in the postoperative hypotonic group.

Monitoring IOP regulation post-vitreoretinal surgery is crucial, particularly in complex cases. The use of SiO may induce IOP fluctuations postoperatively. Ocular hypotony is a common issue following successful repair of complex RRD with SiO endotamponade^(10)^. Post-vitreoretinal surgery, potential causes of ocular hypotony include large RRD leading to increased aqueous outflow into the absorptive compartment of the retinal pigment epithelium and choriocapillaris, cyclodialysis cleft or ciliary body detachment increasing aqueous outflow, ciliary body damage causing reduced secretion and augmented outflow through the uveoscleral pathway, preciliary body membrane fibrosis related with anterior PVR, and chronic traction on the anterior segment due to anterior PVR potentially inducing hypotony^(5)^. To maintain normal IOP levels, aqueous humor production and outflow need to be balanced. “Ciliary shutdown” due to factors such as inflammation or mechanical damage, may be a significant contributor to ocular hypotony following complex RRD. An increased preoperative IOPD between both eyes may indicate the onset of ciliary body dysfunction already present in eyes with complex RRD.

The causes and treatment options for permanent and temporary ocular hypotony post-PPV have been the subject of research for many years. In the literature, ocular hypotony after SiO removal varies between 2% and 23%^(5)^. Various reasons, such as additional relaxing retinotomy, an advanced age, and a low initial IOP in previous ocular surgery, have been suggested for permanent and temporary low IOP post-PPV^(5)^. The changes or tractions because of anterior PVR on the ciliary body cause ocular hypotony^(5^,^10)^. Cyclitic membranes developing from PVR may be blamed as a cause of postoperative persistent ocular hypotonia, although the mechanism is not clear. These membranes may cause secondary ocular hypotonia by closing the ciliary body, leading to ciliochoroidal separation, with or without contraction of the scar tissue they form^(10)^.

Various methods have been reported to avoid ocular hypotony. The Silicone Working Group demonstrated the benefits of SiO tamponade in reducing the incidence of ocular hypotony^(20^,^21)^. Lee et al.^(10)^ reported that clearing the ciliary body membranes with endoscopy-assisted vitrectomy on 15 eyes was an effective treatment for chronic ocular hypotony. Dayani et al.^(16)^ noted that PPV and fluocinolone acetonide implantation with SiO infusion are effective in chronic ocular hypotony. Küçükerdönmez et al.^(22)^ reported that intravitreal or intracameral OVD injections can increase the IOP in eyes with chronic ocular hypotony post-vitreoretinal surgery. Kapur et al.^(23)^ reported that the IOP increased moderately with SiO injection in the 12 eyes of 10 patients who were followed up for chronic ocular hypotony. In our study, we did not remove the SiO (n=26) in patients who could develop ocular hypotony, and we injected an OVD into the anterior chamber when the IOP fell below 6mmHg. We prescribed steroid drops to those patients hourly and monitored their IOP. We reinjected SiO in patients who developed ocular hypotonia after SiO removal.

### Limitations

This study has a few limitations. The first is its retrospective design. Second, ciliary body detachment was not evaluated with ultrasound confirmation and was instead determined only by the surgeon’s observation during surgery. In addition, we only evaluated patients with a PVR grade of C-1 or higher and did not thoroughly examine PVR grading.

In conclusion, prolonged ocular hypotony may result in permanent ocular damage and phthisis bulbi post-PPV. This risk may be higher, especially after complicated RRD surgery with PVR. Identifying factors that may cause ocular hypotony is crucial to avoid these devastating complications. Data suggest that there has been no prior investigation into the disparity in the IOP between the affected eye and the unaffected fellow eye in patients with RRD. At baseline, a relatively low IOP in the nonhypotonic eye compared to its unaffected fellow eye should be a warning for a persistent low IOP.
